# Outcomes of Non-anesthesiologist-Administered Propofol in Pediatric Gastroenterology Procedures

**DOI:** 10.3389/fped.2020.619139

**Published:** 2021-02-02

**Authors:** Frances C. Lee, Karen Queliza, Bruno P. Chumpitazi, Amber P. Rogers, Catherine Seipel, Douglas S. Fishman

**Affiliations:** ^1^Section of Pediatric Gastroenterology, Hepatology, and Nutrition, Texas Children's Hospital, Houston, TX, United States; ^2^Children's Nutrition Research Center, United States Department of Agriculture, Houston, TX, United States; ^3^Department of Anesthesiology, Texas Children's Hospital, Houston, TX, United States; ^4^Department of Pediatric Hospital Medicine, Texas Children's Hospital, Houston, TX, United States

**Keywords:** endoscopy, sedation, pediatric endoscopy, pediatric sedation, propofol

## Abstract

**Background and Aims:** Non-anesthesiologist-administered propofol (NAAP) has been found to have an acceptable safety profile in adult endoscopy, but its use remains controversial and pediatric data is limited. Our aim was to examine the safety and efficacy of NAAP provided by pediatric hospitalists in pediatric endoscopy.

**Methods:** We retrospectively reviewed 929 esophagogastroduodenoscopy (EGD), colonoscopy, and combined EGD/colonoscopy cases in children aged 5–20 years between April 2015 and December 2016 at a large children's hospital. We analyzed the data for adverse events in relation to demographics and anthropometrics, American Society of Anesthesiologists physical classification score, presence of a trainee, comorbid conditions, and procedure time.

**Results:** A total of 929 cases were included of which 496 (53%) were completed with NAAP. Seventeen (3.4%) of NAAP cases had an adverse event including the following: 12 cases of hypoxia, 2 cardiac, and 3 gastrointestinal adverse events. General anesthesia cases had 62 (14.3%) adverse events including the following: 54 cases of hypoxia, 1 cardiac, 7 gastrointestinal, and 1 urologic adverse event. No adverse events in either group required major resuscitation. NAAP vs. general anesthesia had a lower overall adverse event rate (3.4 vs. 14.3%, *p* < 0.0004) and respiratory adverse event rate (2.4% vs. 12.5%, *p* < 0.0004). Overall, cardiac and gastrointestinal adverse event rates between the two groups were comparable. When accounting for all captured factors via logistic regression, both younger age (*P* < 0.001) and general anesthesia (*P* < 0.0001) remained risk factors for an adverse event.

**Conclusion:** The overall adverse event rate of NAAP was low (3.4%) with none requiring major resuscitation or hospitalization. This is comparable to studies of NAAP in adult endoscopy and suggests that NAAP provided by pediatric hospitalists has an acceptable safety profile.

## Introduction

Sedation is important in pediatric endoscopy, as it is necessary for young children to tolerate procedures. Pediatric patients typically require a deeper level of sedation than adult patients in order to avoid discomfort and promote patient cooperation (1). A deeper level of sedation increases the risk of cardiovascular instability (2), and children tend to be at greater risk for airway obstruction given their larger epiglottis and smaller upper airway (1). Outcomes of sedation in pediatric endoscopy are becoming more widely studied. While the methods of sedation vary widely between providers and institutions, recent studies have shown a trend in propofol use in pediatric gastroenterology (3). Propofol is becoming favored as it has limited effect on the gastrointestinal tract, does not increase secretions, and has a rapid onset with a short duration (4, 5). However, propofol has a narrow therapeutic index and can cause respiratory depression and hypotension (4, 5). Due to these effects, propofol use may be restricted to anesthesiologists at some centers.

Recently, there has been a trend toward non-anesthesiologist-administered propofol (NAAP). NAAP has been well-studied and found to be safe in adult endoscopy (6, 7), but studies in children are limited (1, 8–10). In this study, we aim to characterize pediatric patients who underwent non-anesthesiologist administered propofol (NAAP) administered by trained pediatric hospitalists and determine its safety and efficacy. To our knowledge, this is the first study that seeks to examine the outcomes of non-intubated deep sedation administered by a pediatric hospitalist-run sedation program.

## Methods

A retrospective chart review was conducted of all consecutive esophagogastroduodenoscopy (EGD), colonoscopy, or combined EGD/colonoscopy cases between April 2015 and December 2016 at the main campus of Texas Children's Hospital. All procedures included in the study were performed in a GI procedure suite. Complex procedures such as foreign body removals, stricture dilations, motility catheter placements, esophageal variceal surveillance, and banding were excluded, as these procedures are typically ineligible for hospitalist sedation due to a need for deeper anesthesia or airway protection in these cases. Two cases of foreign body removal scheduled non-emergently were included as there was no foreign body visualized or removed. Colonoscopies that led to polypectomies were included in both groups. Data was collected for adverse events related to sedation or anesthesia, including respiratory adverse events such as hypoxia (defined as SpO_2_ <90% by pulse oximetry for longer than 1 min) or need for positive pressure ventilation or intubation, cardiovascular adverse events such as arrhythmias (defined as sustained non-sinus cardiac rhythm seen on cardiac monitors) or symptomatic hypotension (defined as sustained blood pressure <5th percentile for age or <90/50 mmHg for children >10 years), gastrointestinal adverse events such as nausea or vomiting requiring antiemetics, and need for evaluation in the emergency room after the procedure. Events up to 24 h post-procedure that could be attributed to anesthesia-related adverse events were included in the study. Data for adverse events was obtained from vital signs recorded routinely by the anesthesiologist/sedationist during the procedure per a standardized hospital protocol, nursing notes from post-anesthesia care unit (PACU) or telephone calls post-procedure, and documented emergency room (ER) visits. The occurrence of adverse events was analyzed in relation to age, gender, body mass index (BMI), weight, American Society of Anesthesiologists (ASA) physical classification score, presence of a trainee, comorbid condition, and procedure time. IRB approval was obtained for this study.

### Statistical Analyses

Data was analyzed using Fisher exact chi square, Student's *t*-test, Mann–Whitney *U*-test, and logistic regression on IBM SPSS v25. Logistic regression was conducted using the Enter method in SPSS with dependent variable being the presence of an adverse event and independent variables including sedation method, age at time of scope, patient weight percentile, BMI percentile, presence of comorbid condition, presence of a trainee, ASA score, total procedure time, and whether patient was inpatient at time of procedure.

### Hospitalist Sedation and General Anesthesia

All deep sedation cases included in this study were performed by two pediatric hospitalists specializing in sedation. All general anesthesia cases had anesthesia performed and managed by a pediatric anesthesiologist. Patients are referred for deep sedation or general anesthesia by the gastroenterologist performing the procedure. Procedural monitoring in all cases includes the use of pulse oximetry, capnography, blood pressure, and cardiac rhythm monitoring. All deep sedation cases included the use of supplemental oxygen with 2 L nasal cannula (due to end-tidal CO_2_ monitoring affixed to a nasal cannula). For hospitalist sedation, propofol infusion rates are typically 150 mcg/kg/min and decreased as the case progresses. Induction doses average about 2 mg/kg but with titration to effect. Boluses of propofol during the case are on an as-needed basis. Procedural details including supplemental oxygen use and modality, prophylactic medications, and anesthetics are detailed in Supplementary Table 1.

### The Hospitalist Sedation Team

The hospitalist sedation team at Texas Children's Hospital comprises physicians who are board eligible or board certified in critical care medicine, emergency medicine, cardiology with advanced subspecialty training in cardiac intensive care, or pediatrics. Initial training involves working directly with a pediatric anesthesiologist for 5 days in a high-volume, rapid turnover operating room with high risk for airway events, 5 days in the diagnostic imaging suite, at least 20 cases working with a more experienced sedationist or anesthesiologist while leading the sedation, as well as sedation simulation training in emergency resuscitation scenarios. For credentialing, sedationists must score >90% on a deep sedation credentialing exam and must complete hands-on airway management skills training and assessment administered by pediatric anesthesiologists. Physicians must demonstrate competency with a minimum of 10 cases each of the following: deep sedation cases with propofol, inhalational induction with bag-mask ventilation, endotracheal intubations, peripheral IV insertions, oral airway insertions with bag-mask ventilation, and laryngeal mask airway (LMA) insertions. Minimum requirements for re-credentialing include a minimum of 50 cases over a 6-month period and two full sedation shifts per month averaged over 6 months. If individuals do not meet the above requirements, they are required to repeat the training and credentialing process over again. Newly credentialed sedationists work in radiology procedure areas (e.g., MRI, nuclear medicine) for the initial 6–12 months before advancing to sedate in the procedure suite during more invasive procedures, such as EGDs and colonoscopies.

## Results

A total of 1,030 cases were initially reviewed, with 8 cases later excluded for not meeting procedure criteria and 93 cases excluded for patient age <5 or >21 years. Children under age 5 years were excluded from the study as they do not qualify for hospitalist sedation at our institution. Of the 929 included cases, there included a total of 864 patients, with 65 patients that underwent repeat procedures during the review period (see Appendix 2). There were 10 patients who underwent both propofol-based deep sedation and general anesthesia (GA) in separate procedures during the review period. For all 10 patients, no explanation was documented for switching from general anesthesia to deep sedation or vice versa. A total of 496 (53.4%) included cases underwent propofol-based deep sedation administered by pediatric hospitalists with training and experience to administer propofol as part of a hospital-supported sedation team (NAAP). A total of 433 (46.6%) cases underwent general anesthesia (GA). Baseline demographic data for the two groups is shown in Table 1. While there is a slight female predominance in the NAAP group, the gender differences between the two groups are not significant (*p* = 0.066). The two groups also had comparable numbers of each type of procedure (*p* = 0.08). The NAAP group was older in age and had a lower mean BMI percentile, although both groups had similar age and BMI percentile ranges. While both groups had comparable numbers of patients with comorbid conditions, the NAAP group was predominantly ASA 2, while the general anesthesia group had more patients categorized as ASA 3 and 4. The most common comorbid condition in both groups was asthma. The NAAP group had overall shorter average procedure times, as well as anesthesia, room, and PACU stay duration times.

**Table 1:** Demographics of the NAAP and general anesthesia groups for all data and ASA 2-only sub-analysis.

**Demographics for all data**				**Demographics for ASA 2 Only**			
	**NAAP**	**General anesthesia**	***p-*Value**		**NAAP**	**General anesthesia**	***p*-Value**
	**(*n* = 496)**	**(*n* = 433)**			**(*n* = 470)**	**(*n* = 290)**	
Gender			0.066	Gender			0.445
Male	230	227		Male	220	144	
Female	266	206		Female	250	146	
Age				Age			
Mean	13.06 ± 3.6	12.10 ± 3.96	0.002	Mean	13.07 ± 3.5	12.08	
Median	14	12		Median	14	12	
Range	5–20	5–20		Range	5–20	5–20	
IQR	6	6		IQR	6	6	
BMI percentile (%)			0.000	BMI percentile (%)			
Mean	47.8 ± 29.8	54.9 ± 34.2		Mean	47.8 ± 29.7	55.8 ± 33	
Median	45.9	60.4		Median	46.3	61	
Range	0–99.38	0–99.99		Range	0–99.4	0–99.9	
IQR	50.6	65.7		IQR	64.4	60.9	
ASA score			0.000				
ASA 1	5	38					
ASA 2	470	290					
ASA 3	21	103					
ASA 4	0	2					
Comorbid conditions			0.004				
Present	310	309					
None	186	124					
Patient status			0.000	Patient status			0.000
Inpatient	16	49		Inpatient	9	23	
Outpatient	480	384		Outpatient	461	267	
Presence of a trainee			0.000	Presence of a trainee			0.000
Trainee present	86	126		Trainee present	78	86	
No trainee	410	307		No trainee	392	204	
Procedure type			0.080	Procedure type			0.062
EGD	284	223		EGD	275	151	
Colonoscopy	60	57		Colonoscopy	57	30	
EGD/colonoscopy	152	163		EGD/colonoscopy	138	109	
Time (minutes)				Time (minutes)			
Total procedure				Total procedure			
Mean	25.76 ± 21.8	37.2 ± 29.1	0.0004	Mean	24.9 ± 20.5	37.3 ± 29.7	0.0004
Median	15	26		Median	15	24	
Range	4–132	3–140		Range	4–106	3–140	
IQR	30	42		IQR	29	42	
EGD				EGD			
Mean	10.68 ± 7.3	15.7 ± 26.3	0.0004	Mean	10.5 ± 7.1	15.7 ± 11.8	0.0004
Median	11	16		Median	11	16	
Range	4–46	3–140		Range	4–46	3–140	
IQR	8	10		IQR	7	9	
Colon				Colon			
Mean	13.53 ± 19.7	20.2 ± 26.3	0.0004	Mean	12.7 ± 18.7	20.7 ± 27.1	0.0004
Median	31	37		Median	31	39	
Range	11–115	8–124		Range	12–97	12–124	
IQR	19	25		IQR	17	26	
Anesthesia time				Anesthesia time			
Mean	42.3 ± 24.8	61.1 ± 31.9	0.0004	Mean	41.5 ± 23.8	60.7 ± 32.2	0.0004
Median	32	51		Median	32	48	
Range	10–162	14–202		Range	10–162	14–202	
IQR	34.3	44		IQR	34	43	
Total room time				Total room time			
Mean	41.2 ± 24.3	56.31 ± 31.9	0.0004	Mean	40.4 ± 23.2	56.4 ± 32.2	0.0004
Median	32	46		Median	31	45	
Range	13–158	12–200		Range	13–158	12–200	
IQR	34	43		IQR	33	44	
PACU stay duration				PACU stay duration			
Mean	50.7 ± 24.9	55.4 ± 26.3	0.151	Mean	50.8 ± 25.2	55.1 ± 20.6	0.153
Median	46	51		Median	46	52	
Range	17–337	25–358		Range	17–337	25–145	
IQR	19	24		IQR	19	24	

Adverse events comparison between both groups is found in Table 2. Overall, the general anesthesia group had a higher rate of hypoxia and desaturations that lasted longer than 60 s. The NAAP group had nine patients who received positive-pressure ventilation, two patients who received bag mask ventilation, and higher rates of bronchospasm and laryngospasm, although the difference was not significant (*p* = 1.000, *p* = 0.052). One out of the 12 patients in the NAAP group with hypoxia had an LMA placed electively after laryngospasm. The NAAP group had two cardiac adverse events, in the form of a self-resolving wide-complex tachycardia to a heart rate of 205 and one case of syncope shortly after the patient arrived home. The GA group had one cardiac adverse event in the form of PVCs noted during induction. The two groups had comparable rates of cardiac adverse events, and the GA group had slightly more gastrointestinal events. The NAAP group had two patients requiring treatment with antiemetics, and one patient who presented to the emergency room with hematemesis several hours after the procedure. The GA group had seven patients with nausea and emesis receiving treatment with antiemetics, with three emergency room visits for emesis, and one hospital admission for IV fluids in the setting of intractable nausea and vomiting.

**Table 2:** Characterization of adverse events for all data and ASA 2-only sub-analysis.

**Adverse events for all data**				**Adverse events for ASA 2 only**			
	**NAAP**	**GA**	***p*-Value**		**NAAP**	**GA**	***p*-Value**
	**(*n* = 496)**	**(*n* = 433)**			**(*n* = 470)**	**(*n* = 290)**	
**Respiratory**				**Respiratory**			
Hypoxia	12	54	0.0004	Hypoxia	12	26	0.0001
Lowest O_2_ sat	85%	50%		Lowest O_2_ sat (%)	85	62	
Desaturation <90% lasting longer than 1 min	0	11	0.0004	Desaturation <90% lasting longer than 1 min	0	5	0.007
Positive pressure ventilation	9	0	0.005	Positive pressure ventilation	9	0	0.005
Bag mask ventilation	2	0	0.502	Bag mask ventilation			
Bronchospasm	1	0	1.000	Bronchospasm			
Laryngospasm	7	1	0.052	Laryngospasm	7	0	0.037
**Cardiac**				**Cardiac**			
Arrhythmia	1	1	1.000	Arrhythmia	1	1	1.000
Syncope	1	0		Syncope	1	0	
**Gacardiacstrointestinal**				**Gastrointestinal**			
Treatment with antiemetics	2	7	0.06	Treatment with antiemetics	2	6	0.031
Hematemesis	1	0	1.000	Hematemesis	0	0	
**Other**				**Other**			
Urinary retention	0	1	0.466	Urinary retention	0	0	1.000
ER visit	1	3	0.253	ER visit	1	2	
Admission	0	1	0.466	Admission	0	1	

The NAAP group had 17 overall adverse events, giving a rate of 3.4%, and the GA group had 62 total adverse events, with an adverse event rate of 14.4% (Appendix 1). The difference between the overall adverse event rate of the two groups is significant (*p* < 0.0004). The overall respiratory adverse event rate between the two groups was also significant (*p* = 0.034), with the NAAP group having a respiratory adverse event rate of 2.4% and the GA group 12.5%. There was no significant difference between the cardiac, gastrointestinal, and other adverse event rates. A logistic regression shows that there is a significant difference between NAAP and GA, favoring the NAAP group (*p* < 0.0004) (Table 3). The age of the patients is also significant with younger patients having fewer adverse events, especially in the NAAP group. The weight and BMI percentile of the patient, ASA score, presence of a comorbid condition, presence of a trainee, whether the patient was inpatient or outpatient for the procedure, and the total procedure time all did not significantly contribute to the overall adverse event rate (Table 3).

**Table 3:** **(A)** Logistic regression using all data; **(B)** logistic regression with only ASA level 2 patients.

	***B***	**Sig**	**Exp(*B*)**	**95% CI**
**A. Logistic regression using all data**				
NAAP vs. GA	−1.112	0.000	0.329	0.179–0.603
Age at time of scope	−0.119	0.000	0.888	0.832–0.948
Weight %	0.000	0.977	1.00	0.985–1.016
BMI %	0.007	0.417	1.007	0.991–1.023
Presence of comorbid conditions	−0.466	0.164	0.627	0.325–1.210
Presence of trainee	0.514	0.127	1.672	0.864–3.235
ASA score		0.056		
ASA 1	−21.391	0.999	0.000	
ASA 2	−22.292	0.999	0.000	
ASA 3	−21.582	0.999	0.000	
Total procedure time	0.009	0.053	1.009	1.00–1.018
Patient status	−0.589	0.268	0.555	0.196–1.574
Nagelkerke *R*^2^	0.175			
**B. Logistic regression with only ASA level 2 patients**				
NAAP vs. GA	−0.857	0.012	0.424	0.217–0.830
Age at time of scope	−0.137	0.001	0.872	0.804–0.945
Weight %	−0.005	0.598	0.995	0.976–1.014
BMI %	0.013	0.206	1.013	0.993–1.033
Presence of comorbid conditions	−0.459	0.208	0.632	0.309–1.291
Presence of trainee	0.263	0.507	1.3	0.599–2.823
Total procedure time	0.014	0.011	1.014	1.003–1.025
Patient status	−0.589	0.268	0.555	0.304–4.189
Nagelkerke *R*^2^	0.129			

The adverse events in both groups were classified as pre-, intra-, or post-procedure (Supplementary Table 2). The NAAP group had 13 intra-procedural adverse events including 12 cases of hypoxia and 1 case of arrhythmia; 4 post-procedural adverse events including 1 case of syncope, 2 cases of nausea/vomiting, and 1 case of hematemesis; and no pre-procedural events. The GA group had 1 pre-procedural adverse event (a case of arrhythmia during induction), 54 intra-procedural adverse events (all cases of hypoxia), and 8 post-procedural events in the form of 7 cases of nausea/vomiting and 1 case of urinary retention.

The adverse events were further classified using the common terminology criteria for adverse events (11) (Supplementary Table 2). This system grades events by severity with grade 1 being mild, grade 3 being severe, and grade 5 being death (11). Overall, the majority of the adverse events in both groups were grade 1 or mild. There was one grade 3 event in the general anesthesia group, in the form of an admission to the hospital lasting longer than 24 h for intractable vomiting requiring IV fluids.

The post-procedural events were classified using a system developed by Kramer and Narkewicz (12) (Supplementary Table 2). Grade 1 is a mild event requiring supportive care or telephone management, grade 2 is an adverse event requiring ER visit or unanticipated evaluation by a physician, and grade 3 is an admission (12). The majority of the post-endoscopy events in the general anesthesia group were grade 1, while the deep sedation group was evenly distributed between grade 1 and grade 2.

A sub-analysis was conducted comparing only ASA level 2 patients between the two groups, as the NAAP group was predominantly ASA level 2 (Table 1). The NAAP group has a slightly higher mean and median age and shorter procedure, anesthesia, room, and PACU times. The number of patients who are inpatient vs. outpatient, as well as the presence of a trainee, remains significantly different between the two groups. The number of adverse events in the NAAP group remains unchanged (Table 2), indicating that all the adverse events took place in ASA level 2 patients in that group. The GA group had fewer cases of hypoxia (*n* = 26), however still significantly more than the NAAP group (*p* <0.0004). There is one less gastrointestinal event in the GA group. The number of cardiac events in both groups is unchanged. The overall adverse event rate of the NAAP group remains at 3.4%, and the overall adverse event rate of the GA group using only ASA 2 patients is 11% (*p* < 0.0004, Appendix 1). The difference in the respiratory adverse event rates of the two groups is still significant (*p* < 0.0004), with the respiratory adverse event rate of the NAAP group being 1.7% while the respiratory adverse event rate of the GA group is 9.0%. The difference in the gastrointestinal event rates between the two groups is now significant (*p* = 0.031) while the rates of cardiac adverse events remain insignificant. The logistic regression with only ASA 2 patients continues to favor the NAAP group over the GA group (*p* = 0.012), with age being significant and favoring the NAAP group (*p* = 0.001). The total procedure time is significant with longer procedure times having fewer adverse events (*p* = 0.011). The weight and BMI percentile of the patient, ASA score, presence of a comorbid condition, presence of a trainee, whether the patient was inpatient or outpatient for the procedure did not significantly contribute to the overall adverse event rate (Table 3).

We conducted propensity score matching in SPSS for age, BMI, weight, ASA, presence of a comorbid condition, presence of a trainee, total procedure time, and patient status (inpatient vs. outpatient). This resulted in 158 patients, with 87 in the deep sedation group and 71 in the GA group. Differences in gender (*p* = 0.523), presence of a comorbid condition (*p* = 0.576), presence of a trainee (*p* = 0.062), weight (*p* = 0.352), and gender (*p* = 0.523) were not statistically significant. ASA score (*p* < 0.001) and patient status (*p* = 0.017) remained significantly different between the two groups. Logistic regression of this data set had a Nagelkerke *R*^2^ of 0.582, and only the method of sedation was significant (*p* < 0.001, *B* = −3.686, 95% CI 0.008–0.084) favoring deep sedation over GA.

## Discussion

Sedation is integral to the success of pediatric endoscopies, as it ensures patient comfort and cooperation. However, in pediatric endoscopy procedures, complications that arise from sedation can occur more frequently than complications from the endoscopic procedure itself (13). Propofol-based sedation is on the rise, as is the use of NAAP. NAAP by pediatric hospitalists has not been widely studied or characterized in pediatric endoscopy. To our knowledge, our study is the first to examine the safety and efficacy of NAAP by pediatric hospitalists in pediatric endoscopy. We found that NAAP by pediatric hospitalists (vs. general anesthesia) for pediatric endoscopy resulted in fewer adverse events. This difference in adverse events between NAAP and general anesthesia persisted even when accounting for known risk factors such as age and ASA classification. These findings suggest NAAP for pediatric endoscopy has an acceptable safety profile.

Our NAAP pediatric endoscopy findings appear to complement other available pediatric non-anesthesiologist-administered anesthesia studies—the majority of which were completed in other hospital settings. Khalila et al. (14) examined 1,190 pediatric endoscopic procedures (all ASA 1 or 2), with NAAP by the pediatric gastroenterologist performing the procedure, and found a 0.7% adverse event rate, comparable to adult studies in which sedation is performed by the adult gastroenterologist. A study by Hertzog et al. examined rates of adverse events of non-anesthesiologist-provided sedation in pediatric procedural sedation, including approximately 2,100 cases by pediatricians (15). The study, which was not limited to propofol-based sedation and included 6.1% GI procedures, found an overall adverse event rate of 5.3% (15), but did not specify adverse event rate by type of provider, as that was not the goal of their study.

Jain et al. (8) compared propofol-based deep sedation by pediatric critical care or emergency medicine providers to general anesthesia for children undergoing cardiac MRI. They had a 3.4% adverse event rate in their deep sedation group (compared to a 4.7% adverse event rate in the general anesthesia group), with adverse events including airway obstruction requiring nasopharyngeal or oral airway, hypotension requiring IV fluids, desaturations requiring PPV, and excessive secretions requiring suctioning (8). A study conducted by Rajasekaran et al. (1) examined the safety of deep sedation in pediatric EGDs performed by an intensivist-run sedation program. The study found a 3% overall complication rate for propofol-based sedation in 2,325 pediatric EGDs over the course of 4 years (1).

We found respiratory adverse events to be most common in both the NAAP and general anesthesia groups. Similar to the study by Hertzog et al., the most common adverse events in both the NAAP and GA group in our study was hypoxia as measured by pulse oximetry (15). The NAAP group had a lower rate of respiratory adverse events when compared to the GA group. This is significant as hypoxemia is thought to be a significant contributor to cardiopulmonary complications during endoscopy (16). It is possible that the differences in respiratory adverse events between the two groups are due to the use of airway devices (intubation or LMA) that can be associated with increased risk of respiratory events by the GA group. Other considerations include differences in equipment in the GA room and hospitalist sedation rooms and the method of data collection, which may have included artifactual data (such as false pulse oximetry readings due to patient movement or probe misplacement) in addition to adverse events. The adverse event rate of the general anesthesia group in our study was 14.3%, which is higher than other similar studies in the past. This is most likely due to the differences in study parameters, such as the definition of hypoxia (the SpO_2_ reading cut-off), and the inclusion of post-procedure events.

Our study also found that procedure times were lower in those undergoing NAAP. Rajasekaran et al. also compared procedure times of 549 deep sedation patients to 13 general anesthesia patients and found that deep sedation had shorter length of sedation times (22.1 min) when compared to anesthesia patients (38.3) (1). However, the difference was not significant. In our study, the mean total procedure, EGD, colonoscopy, and total anesthesia times for the deep sedation group were significantly shorter in the deep sedation group compared to the anesthesia group. The difference in total procedure times between the two groups may be influenced by the presence of trainees, who are more likely to be assigned to general anesthesia procedures. There was no difference in the PACU stay times between the two groups; however, the PACU stay duration was highly affected by factors such as the availability of transportation for the patient's families or the availability of an inpatient bed for patients who are being admitted to the hospital post-procedure. Notably, in our study, the total procedure time in the sub-analysis with only ASA 2 patients becomes significant with longer procedure times having fewer adverse events (*p* = 0.011), favoring the GA group. Likely, this is secondary to adverse events leading to abbreviated procedures or the hastened completion of those procedures.

Limitations to this study include its retrospective nature, and as such, adverse event reporting was at the discretion of the medical team. However, we note that certain events such as hypoxia are uniformly captured prospectively during procedures, and all care was documented per standard medical care in our medical system. Another limitation includes differences in the age and ASA scores between NAAP and general anesthesia groups of patients. We accounted for this difference both by focusing on patients with ASA 2 in a sub-analysis and by accounting for both age and ASA status in our regression analyses. Both the sub-analysis and regression analyses continued to demonstrate higher adverse events in those with general anesthesia. We also note the possibility of bias in the referral process in which patients are assigned to general anesthesia or hospitalist sedation. This bias occurs at the level of the gastroenterologist, who is referring patients for hospitalist sedation or anesthesia, as well as the anesthesiologist/sedationist's discretion as to the method of sedation that they deem most appropriate for the patient. This may lead to healthier patients being selected for hospitalist sedation over general anesthesia. Patients with higher ASA scores and who were inpatient were more likely to receive general anesthesia. This was reflected in the propensity score matching calculations.

Future studies in this area are needed. Future directions can include the investigation of differences in cost between NAAP and general anesthesia. Rajasekaran et al. found that deep sedation was significantly more cost effective than general anesthesia; however, the study was only able to directly compare 13 general anesthesia cases to 549 deep sedation cases due to the majority of the general anesthesia cases being combined procedures. Other areas of investigation could include patient satisfaction, evaluating the difference between the length of time off work or school post-procedure between the two groups of patients, as well as direct comparison of outcomes between propofol-based deep sedation by pediatric hospitalists and anesthesiologists.

The pediatric hospitalists who provided the propofol-based deep sedation in this study were all trained in accordance to ASGE recommendations (7). The overall adverse event rate of NAAP in our study is comparable to the results of adult studies and similar pediatric studies. In our study, NAAP had a lower overall adverse event rate than general anesthesia cases and a lower overall respiratory adverse event rate. The rates of cardiac and gastrointestinal adverse events between the two groups are similar. Patients who are ASA 3 and above, or otherwise at high risk for anesthesia complications, should still be referred for general anesthesia. The results of our study suggest that NAAP deep sedation by a pediatric hospitalist during pediatric EGD, colonoscopy, and EGD/colonoscopy is safe and effective. NAAP by a pediatric hospitalist-run sedation team is an example of multidisciplinary collaboration to produce high-quality care for pediatric patients.

## Data Availability Statement

The original contributions presented in the study are included in the article/Supplementary Materials, further inquiries can be directed to the corresponding author/s.

## Ethics Statement

The studies involving human participants were reviewed and approved by Baylor College of Medicine. Written informed consent from the participants' legal guardian/next of kin was not required to participate in this study in accordance with the national legislation and the institutional requirements.

## Author Contributions

FL: data collection, data analysis, and manuscript writing. KQ: data collection and manuscript editing. BC: data analysis and manuscript editing. AR: experimental design and manuscript editing. CS: manuscript editing. DF: experimental design, data analysis, manuscript writing, and manuscript editing. All authors contributed to the article and approved the submitted version.

## Conflict of Interest

The authors declare that the research was conducted in the absence of any commercial or financial relationships that could be construed as a potential conflict of interest.
