# Supplementary material for: Outcomes of Non-anesthesiologist-Administered Propofol in Pediatric Gastroenterology Procedures
Source: Front Pediatr. 2021 Feb 2;8:619139. doi: 10.3389/fped.2020.619139 (PMC7885908; doi:10.3389/fped.2020.619139)
Supplement: Supplementary file 1 [file Table_1.docx]

| **Procedural Data (All Data)** | | | | **Procedural Data (ASA 2 only)** | | | |
| --- | --- | --- | --- | --- | --- | --- | --- |
|  | **NAAP (n = 496)** | **GA (n=433)** | **p-value** |  | **NAAP (n=470)** | **General Anesthesia (n=290)** | **p-value** |
| **Oxygen** |  |  |  | **Oxygen** |  |  |  |
| **Nasal cannula** | 496  (100%) | 48  (11.1%) | 0.000 | **Nasal cannula** | 470  (100%) | 35  (13%) | 0.000 |
| **ETT/LMA** | 0 | 384  (88.7%) | 0.000 | **ETT/LMA** | 0 | 253  (93.7%) | 0.000 |
| **Dexamethasone** | 2  (0.4%) | 212  (50.0%) | 0.000 | **Dexamethasone** | 2  (0.4%) | 149  (51.4%) | 0.000 |
| **Prophylactic Antiemetics** | 2  (0.4%) | 341  (78.8%) | 0.000 | **Prophylactic Antiemetics** | 2  (0.4%) | 229  (84.8%) | 0.000 |
|  |  |  |  |  |  |  |  |
| **Anesthetics** |  |  |  | **Anesthetics** |  |  |  |
| **Propofol** | 496  (100%) | 388  (89.6%) | 0.000 | **Propofol** | 470  (100%) | 264  (91%) | 0.000 |
| **Fentanyl/Versed** | 490  (98.8%) | 192  (44.3%) | 0.000 | **Fentanyl/Versed** | 464  (98.7%) | 123  (42.4%) | 0.000 |
| **Dexmedetomidine** | 145  (29.2%) | 137  (31.6%) | 0.426 | **Dexmedetomidine** | 141  (30%) | 98  (33.8%) | 0.274 |
| **Nitrous oxide** | 9  (1.8%) | 79  (18.2%) | 0.000 | **Nitrous oxide** | 9  (1.9%) | 55  (19%) | 0.000 |
| **Sevoflurane** | 0 | 401  (92.6%) | 0.000 | **Sevoflurane** | 0 | 272  (93.8%) | 0.000 |
| **Desflurane** | 0 | 49  (11.3%) | 0.000 | **Desflurane** | 0 | 27  (9.3%) | 0.000 |

Supplemental Table 1. Additional Procedural Data
