# Supplementary material for: Outcomes of Non-anesthesiologist-Administered Propofol in Pediatric Gastroenterology Procedures
Source: Front Pediatr. 2021 Feb 2;8:619139. doi: 10.3389/fped.2020.619139 (PMC7885908; doi:10.3389/fped.2020.619139)
Supplement: Supplementary file 2 [file Table_2.docx]

| **Timing of Adverse Event** | **Pre-procedure** | **Intra-procedure** | **Post-procedure** |
| --- | --- | --- | --- |
| **MAC** | 0 | 13 | 4 |
| **GA** | 1 | 54 | 8 |
| **CTCAE Grade** | **1** | **2** | **3** |
| **MAC** | 8 | 9 | 0 |
| **GA** | 60 | 2 | 1 |
| **Kramer Classification** | **1** | **2** | **3** |
| **MAC** | 1 | 2 | 0 |
| **GA** | 6 | 2 | 1 |

Supplemental Table 2. Timing of adverse events; CTCAE grade; Classification of adverse events using system developed by Drs. Kramer and Narkewicz
