# Supplementary material for: Outcomes of Non-anesthesiologist-Administered Propofol in Pediatric Gastroenterology Procedures
Source: Front Pediatr. 2021 Feb 2;8:619139. doi: 10.3389/fped.2020.619139 (PMC7885908; doi:10.3389/fped.2020.619139)
Supplement: Supplementary file 3 [file Data_Sheet_1.docx]

| **Adverse Event Rate for All Data** | | | | **Adverse Event Rate for ASA 2 Only** | | | |
| --- | --- | --- | --- | --- | --- | --- | --- |
| **Adverse event rate** | **NAAP (n = 496)** | **GA (n=433)** | **p-value** | **Adverse event rate** | **NAAP (n=470)** | **General Anesthesia (n=290)** | **p-value** |
| **Overall** | 17 (3.4%) | 63 (14.5%) | 0.0004 | **Overall** | 16  (3.4%) | 33  (11.4%) | 0.000 |
|  |  |  |  |  |  |  |  |
| **Respiratory** | 12 (2.4%) | 54 (12.5%) | 0.034 | **Respiratory** | 8  (1.7%) | 26  (9.0%) | 0.000 |
|  |  |  |  |  |  |  |  |
| **Cardiac** | 2 (0.4%) | 1 (0.2%) | 0.650 | **Cardiac** | 2  (0.4%) | 1  (0.3%) | 0.863 |
|  |  |  |  |  |  |  |  |
| **Gastrointestinal** | 3 (0.6%) | 7 (1.6%) | 0.136 | **Gastrointestinal** | 2  (0.4%) | 6  (2.1%) | 0.031 |
|  |  |  |  |  |  |  |  |
| **Other** | 0 (0.0%) | 1 (0.2%) | 0.466 | **Other** | 0  (0.0%) | 0  (0.0%) | 1.000 |

Datasheet 1. Adverse event rate for all data and ASA 2-only sub-analysis
