# Supplementary material for: Outcomes of Non-anesthesiologist-Administered Propofol in Pediatric Gastroenterology Procedures
Source: Front Pediatr. 2021 Feb 2;8:619139. doi: 10.3389/fped.2020.619139 (PMC7885908; doi:10.3389/fped.2020.619139)
Supplement: Supplementary file 4 [file Data_Sheet_2.docx]

**Datasheet 2**

1030 cases reviewed

93 cases excluded for age <5 or > 21

8 cases excluded

929 cases included, 864 patients, 65 repeat in both groups
